# Review of PIP2 in Cellular Signaling, Functions and Diseases

**DOI:** 10.3390/ijms21218342

**Published:** 2020-11-06

**Authors:** Kalpana Mandal

**Affiliations:** Institute for Medicine and Engineering, University of Pennsylvania, Philadelphia, PA 19104, USA; mandalk@pennmedicine.upenn.edu

**Keywords:** Phosphoinositides, PIP2, membrane dynamics, actin, intracellular trafficking, focal adhesion, diseases

## Abstract

Phosphoinositides play a crucial role in regulating many cellular functions, such as actin dynamics, signaling, intracellular trafficking, membrane dynamics, and cell–matrix adhesion. Central to this process is phosphatidylinositol bisphosphate (PIP2). The levels of PIP2 in the membrane are rapidly altered by the activity of phosphoinositide-directed kinases and phosphatases, and it binds to dozens of different intracellular proteins. Despite the vast literature dedicated to understanding the regulation of PIP2 in cells over past 30 years, much remains to be learned about its cellular functions. In this review, we focus on past and recent exciting results on different molecular mechanisms that regulate cellular functions by binding of specific proteins to PIP2 or by stabilizing phosphoinositide pools in different cellular compartments. Moreover, this review summarizes recent findings that implicate dysregulation of PIP2 in many diseases

## 1. Introduction

**Figure 1:**
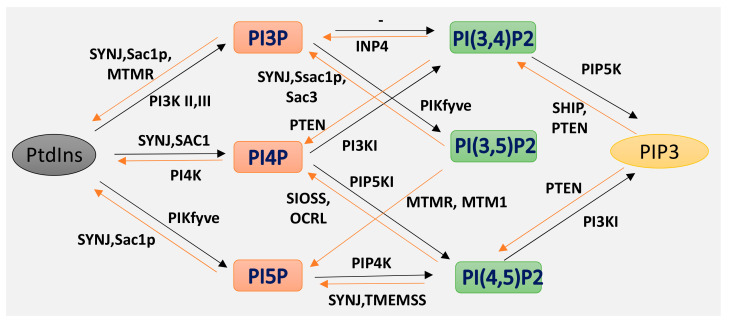
Isoforms of phosphoinositides. By the action of PIK and phosphatase, phosphatidylinositol (PtdIns) and the three isoforms of PIP2 are formed, as indicated here. The specific action of PI3K I, II III and of the 3-phosphatases are also illustrated.

Phosphoinositides (PPIs), are inositol-containing glycerophospholipids bearing variable numbers of phosphate groups on their headgroups [1]. PPIs are multifaceted molecules that have recently become an interesting player in regulating cell function due to their involvement in cellular functions such as actin dynamics, membrane trafficking, regulation of transmembrane proteins and signal transduction [2,3,4,5,6,7,8]. Although the total amount of PPIs in eukaryotic cell membranes is low, they play critical roles in cellular dynamics by regulating multiprotein complexes [9,10]. The spatiotemporal regulation of PPI-mediated biological processes is achieved by interconversion (Figure 1) of the phosphorylation states of PPIs by specific kinases and phosphatases, followed by recruitment of PPI-specific effectors. Inter-conversion of the phosphate groups is spatially controlled by phosphoinositide-metabolizing kinases and phosphatases as required for cellular functions [11,12]. PPIs generate seven possible isoforms by phosphorylating the inositol ring at positions 3, 4, and 5. Three isoforms of PPIs with two phosphate groups connected to the inositol ring, phosphatidylinositol-(4,5)-bisphosphate (PI(4, 5)P_2_), phosphatidylinositol-(3,5)-bisphosphate (PI(3, 5)P_2_) and phosphatidylinositol-(3,4)-bisphosphate (PI(3, 4)P_2_) are the focus of this review. PI(3,4)P2 and PI(3,5)P2 are produced by the phosphorylation of PI3P, and PI(4,5)P2 is produced by the phosphorylation of PI4P or PI5P. The synthesis of PIP2 by phosphorylation of PI5P is regulated by PIP4K, which is one of the less studied pathways. In addition, PIP4K is able to phosphorylate PI3P, but only able to alter PI5P levels in vivo [13] (PI(4, 5)P_2_) can be further phosphorylated to (PI(3,4,5)P_3_) and (PI(3,4,5)P_3_) is converted to (PI(4,5)P_2_) by the enzyme phosphate and tensin homolog deleted from chromosome 10 (PTEN) [14]. PIP3 stimulates the activity of PDK1 and phosphorylates Akt [15,16]. This PIP3/Akt pathway is intensively studied and regulates many crucial processes in cells. PIP_3_ and PTEN have been the subjects of excellent recent reviews, and the focus here is on PIP_2_ [7].

Phosphoinositides control intracellular trafficking, membrane dynamics and cytoskeletal organization by interacting with many different proteins [17,18,19]. PIP_2_ regulates other membrane phospholipids and their signaling functions [7,17]. The major roles it plays in the cell membrane include cytoskeletal linkage, regulation of ion channels, and intracellular trafficking [20]. PI dynamics and mechanism are precisely controlled by kinase and phosphatase [21,22]. Recent studies showed the direct implication of these enzymes in diseases including liver cancer, glioblastoma or neurodegeneration [7,11,23]. Thus, many studies target phosphoinositide kinase inhibitors for pathological studies.

In this review we summarize the recent development in understanding the role of PIP_2_ in cellular function and signaling. We first discuss the effect of PIP_2_ on actin binding proteins, addressing the mechanism of the actin cytoskeletal dynamics such as polymerization or depolymerization of the filamentous network or the coupling to membrane to generate forces. Next, we outline the role of PIP_2_ in membrane dynamics. We summarized how the membrane organization depends upon PIP_2_ in the presence of ions or transmembrane proteins that are sensitive to membrane curvature. We discuss how clathrin coated pits interact with adaptor proteins during the endocytosis process, which is facilitated by PIP_2_. Finally, we discuss the role of PIP_2_ in cell signaling and diseases.

## 2. PIP_2_ in Actin Dynamics

Cytoskeletal dynamics play an important role in many cellular functions such as force generation, intracellular transport, or migration [24,25,26,27]. Actin forms the network inside the cell which is the most responsible for cellular architecture providing the cell a mechanical scaffold [24,27,28]. Accumulated evidence suggests that membrane PIP_2_ regulates the function of many acting binding proteins including formin, gelsolin, cofilin, profilin, filamin, WASP, ezrin, α- actinin, and others, which control the dynamical organization of the actin network [9,29,30,31,32,33]. PIP_2_ mostly inactivates actin binding proteins that inhibit actin polymerization and activates proteins which promote filamentous assembly [30,34]. Proteins bind to PIPs via numerus different structures, including the pleckstrin homology (PH) domain of phospholipase C-delta1, the Gag precursor protein Pr55 of HIV-1, phox homology (PX), C2, SH2, protein tyrosine binding, FYVE, PHD, GRAM, BAR, and espin N-terminal homology (ENTH)/ANTH domains, forming a large family of domains collectively [35,36].

**Figure 2:**
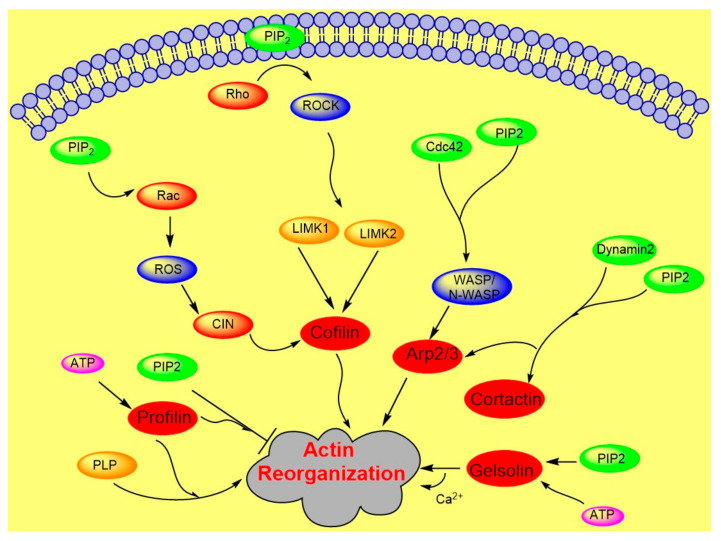
Role of PIP2 in actin dynamics either by promoting polymerization or inhibiting severing. The figure summarizes gelsolin, profilin, cofilin, Arp2/3, and WASP dynamics in coordination with Rho- ROCK and Rac pathways.

Actin polymerization is regulated by a variety of actin binding proteins [28,37]. Actin dynamics depend upon the continuous attachment of G- actin at the barbed (+) end and dissociation at the pointed (−) end, and that defines the filament length [38]. Cofilin is an actin binding protein that binds to both F-actin and G-actin and is a severing protein responsible for actin depolymerization (Figure 2) [38,39,40]. ADF/ cofilin binds to PIP_2_ through a multivalent mechanism and the dissociation of ADF/cofilin to actin filament can accurately be regulated by changing PIP_2_ density at the cell membrane [41]. One study found that cofilin binds to PIP_2_ via a specific pocket which is pH dependent [42]. However, this result contrasts with recent finding showing that cofilin interaction with PIP_2_ is not pH dependent, but the interaction of profilin with membrane, actin and multiple PIP_2_ headgroup (clustering) is affected somewhat when pH is increased [41]. Cofilin’s activity depends on phosphorylation, which is regulated by Rho-GTPase and LIM kinase (LIMK) and by binding PPIs [43]. The rho-family small GTPases, Rho, Rac, and Cdc42, play a central role in regulating actin reorganization through their various downstream effectors [44]. LIMK1 and LIMK2 are activated by the GTPase-dependent protein kinases ROCK and PAK1 by phosphorylation of Thr508 and Thr-505, respectively, in the activation loop of the kinase domain [4,45]. LIMK1 and LIMK2 both regulate actin cytoskeletal reorganization by phosphorylating and inactivating cofilin/ADF [4,6]. Hence, cofilin is regulated by the signals from both the Rho and Rac pathways. Epidermal growth factor (EGF) induces sudden loss of PIP_2_ in membrane that activates local cofilin pool in membrane in carcinoma [46]. These altogether lead to a dramatic turnover of actin monomers (Figure 2).

Gelsolin is another actin severing and capping protein which binds to the barbed end of actin filaments [47]. The barbed end of the filament capped by gelsolin becomes available again through the binding of phosphatidylinositol lipids, such as PIP_2_, leading to filament elongation. Three PIP_2_ binding sites for gelsolin have been characterized. Two of the binding sites compete with F-actin and G-actin sites [5,48]. Thus, the severing function of gelsolin can be inhibited by PIP_2_ [5,47]. Gelsolin can bind to the cell membrane by PIP_2_ which abrogates the gelsolin interaction with actin. Not only does the amount of phosphoinositide alter the free or actin-bound-gelsolin in cells, but also the lateral distribution of PIP_2_ controls inactivation of gelsolin [49,50]. Recent studies showed that ATP competes with PIP_2_ to bind with gelsolin [51,52]. Interaction of gelsolin with PIP_2_ can be abrogated chemically in vitro by including profilin which is a competing PIP_2_ binding protein [48]. PIP_2_ binding to the gelsolin family of capping proteins is enhanced by calcium ions [47]. Ca^2+^ potentiates gelsolin’s binding to the end of the filament and promotes the polymerization of monomeric actin into filaments [29,48]. Antibacterial activity of rhodamine B (RhB)-conjugated peptides based on the PIP_2_ binding site of gelsolin, which are cell membrane-permeant, has been shown to kill microorganisms, such as *Escherichia*, *Pseudomonas aeruginosa*, and *Streptococcus pneumoniae* [53,54].

Another important PPI-sensitive player for actin dynamics is the Arp2/3 complex, which regulates nucleation and branching of actin filaments. Lateral organization of PIP_2_ in the lipid bilayer regulates nucleation [55]. Arp2/3 is primarily activated by the Wiskott-Aldrich syndrome protein (WASP) family, multidomain proteins, and PIP_2_ promotes this activation (Figure 2). WASP family proteins integrate PIP_2_ and other signals to regulate cytoskeletal response through the Arp2/3 complex. Moreover, PIP_2_ interaction with PH domain of WASP regulates the stabilization of WASP at the membrane. In Xenopus egg extracts N-WASP interacts with Cdc42, which is a small GTPase protein of the Rho family and is required for actin polymerization. An increase in N-WASP activity is coordinated by Cdc42 and PIP_2_ synergistically [34,56,57].

Profilin is another essential actin regulatory protein which interacts with many other proteins [58]. Decreased of profilin1 expression increases cellular motility by regulating PIP_2_ along with lammellipodin accumulation at the cell leading edge [59]. An in vitro study showed that profilin, isolated from platelets, binds to PIP_2_ along with other phospholipids in lipid bilayers [60]. Profilin regulates tyrosine kinase-coupled PIP_2_ hydrolysis [61]. PLC-γ1 hydrolyzes profilin-bound PIP_2_ by competing the inhibitory effect of profilin [60]. Profilin binds to G-actin and increases the ATP binding to actin [61]. This leads to ATP-actin binding at (+) end of filamentous actin. Profilin binding to actin competes with binding of PIP_2_. Profilin interacts with actin and poly-L-proline (PLP) stretches, which is essential for profilin function in fission yeast [62]. Profilin binds to both PLP and actin monomers simultaneously [7]. In addition, profilin binds to the cell membrane by PI(4,5)P_2_, which prevents actin and PLP interaction [7,58].

A large body of literature shows that PIP_2_ turnover regulates the activity of both gelsolin and profilin. It is clear by now that phosphoinositides and these actin binding proteins interact. However, the mechanism at the molecular level remains elusive. A recent study focuses on different actin binding proteins such as mDia2, N-WASP and gelsolin interaction with PIP_2_ at the membrane by using molecular dynamics simulation and experimental approaches. The study showed that the cholesterol and PIP_2_ distribution alters the interaction between actin binding protein and PIP_2_. With large unilamellar vesicle containing PIP_2_, a multivalent binding model showed that PIP_2_ activates mDia2 and NWASP to nucleate straight and branched actin filaments, respectively, but inhibits gelsolin’s ability to cap the fast-growing barbed end of F-actin or to sever the actin filament [49]. Cortactin is also an actin associated protein that can bind and regulate Arp2/3 and N-WASP [63]. Cortactin mutant cells show reduced binding of Arp2/3 complex or dynamin2 to actin. By performing in vitro experiments it is shown that dynamin2 enhances the nucleation of actin by Arp2/3 and cortactin in PIP_2_ containing vesicles [64].

## 3. PIP_2_ in Adhesion Dynamics

PIP_2_ binds to many focal adhesion proteins, such as vinculin, talin, and the focal adhesion kinase FAK. PIP_2_ serves as linkage to focal adhesion and actin binding proteins. There are actin binding proteins such as α-actinin, ezrin or filamin which also bind to focal adhesions. A synthetic peptide of α-actinin inhibits PLC-γ1 and PLC-δ1 activity and inhibition is induced by PIP_2_ competition [40]. PIP_2_ binding to α-actinin is inhibited by the treatment of cells with platelet derived growth factor, resulting in actin depolymerization. A recent study showed that the architecture of α-actinin-2 and 3 provides a suitable spatial orientation platform for PIP_2_ bonding by performing molecular dynamics (MD) simulations [65]. In smooth muscle in which α-actinin was discovered, PIP_2_ is found in large amounts which facilitates gelation of actin [66,67]. The length of smooth muscle depends upon the PIP_2_ synthesis, which regulates inositol phospholipid turnover [68]. Filamin A is another crosslinker protein which forms contacts between focal adhesions and F-actin. Filamin is associated to the cell membrane by β integrins. PIP_2_ bound to filamin A inhibits the gel formation of actin. Filamin has three isoforms called FLNa, FLNb, and FLNc. FLNa is recruited by CD28 followed by lipid raft accumulation at the immunological synapsis in T lymphocyte activation. PIP_2_ is essential for the clustering of lipid raft [69]. Ezrin is one of the ERM (ezrin, radixin, moesin) family proteins, which also forms linkages between the cellular membrane and cytoskeleton. Ezrin exists in both active and inactive states within cells. PIP_2_ activates Ezrin by binding with it and becomes available for phosphorylation by Rho-kinase and many PKC isoforms [70]. Neutron scattering experiment showed for the first-time, the conformational changes of ezrin when it simultaneously binds to PIP_2_ and F-actin [71].

Focal adhesion kinase (FAK) is a protein tyrosine kinase implicated in many signaling pathways to regulate cellular functions including migration. When a cell binds to the extracellular matrix (ECM), FAK is recruited to focal adhesion (FA) sites and undergoes conformational change, which is activated by phospholipids such as PIP_2_ by unblocking the FERM domain and kinase domain. Simulation results show that FAK transiently binds to PIP_2_ through electrostatic interactions [72]. Molecular dynamics simulation and fluorescence resonance energy transfer (FRET) experiments both showed that FAK binding to ATP decreases the FRET signal confirming that the PIP_2_ binding acts in the reverse direction [73,74]. Phosphatidylinositol 4-phosphate 5-kinase type I*γ* (PIP5KI*γ*) is required for efficient FAK activation and generates PIP_2_ locally in FAs by PIP5KI*γ*. Thus, PIP_2_ is a strong mediator in integrin-FAK signaling pathways [73].

**Figure 3:**
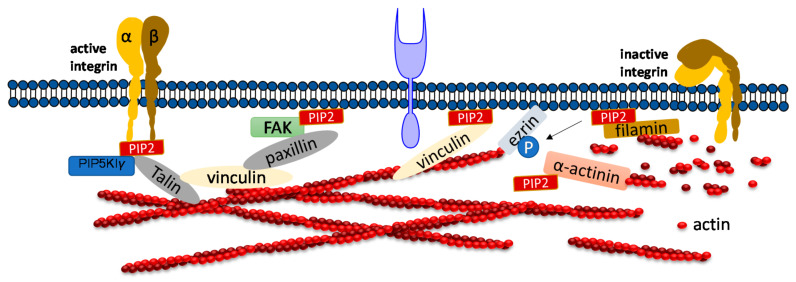
Role of PIP_2_ in regulating focal adhesion assembly. Depiction of adhesion molecules talin, vinculin, ezrin, filamin and a-actinin. PIP_2_ synergistically binds to both talin and integrin and activates both of them. Talin binds directly to actin or activates vinculin and facilitates its binding to actin. PIP_2_ also binds to FERM domain of FAK and binds to vinculin via paxillin. PIP_2_ negatively regulates cross-linking activity of filamin and the actin bundle formation mediated by α-actinin.

Talin plays a crucial role in activating integrins [75,76]. Within the cytosol talin is in an inactivated form, where its C-terminal rod domain binds to the N-terminal head domain. Many pathways lead to disruption of the interaction between talin’s C-terminal and N-terminal including binding with PIP5KI*γ* which generates PIP_2_ from PI4P [77]. Ye et al. delineate a detailed account of PIP_2_ in activating talin by using FRET. They showed interaction of talin with lipid bilayers is altered by PIP_2_ [78]. The FERM domain of talin-1 binds to the cytosolic domain of β_3_ -integrin weakly (Figure 3). However, the interaction affinity increases three-fold when it synergistically binds to acidic PIP_2_ [8,79,80]. Membrane bound talin recruits and activates vinculin. Vinculin localizes at the adhesion complex and interacts with PIP_2_ to associate with the membrane (Figure 3) [81]. Simulation data shows that PIP_2_ is not required for vinculin localization at FAs but is needed for the activation of FA turnover during mechanotransduction processes [81]. Other studies mentioned that PIP_2_ is required for FA formation and vinculin phosphorylation and trafficking [82].

## 4. PIP_2_ in Membrane Dynamics and Organization

Phosphoinositides are minor component of the lipid bilayer that forms the plasma membrane, constituting 1% of total cellular phospholipid. Eukaryotic cell plasma membranes maintain a balanced composition of sterols, phospho- and sphingo- lipids that is distinct from other cellular membranes, which is required for cellular integrity. All seven PIPs are spatially localized uniquely in the plasma membrane. However, PIP_2_ is the most abundant among all seven species of PIPs. Many PIP_2_ binding proteins are characterized as high affinity ligands for these lipids to regulate signaling [83] and are activated by agonists for numerous cell surface receptors [33]. Several studies reported that PIP_2_ is highly enriched in the plasma membrane within segregated domains with an approximate size of 73 nm by showing PC12 cell staining with anti-PIP_2_ antibody and high-resolution STED imaging [84,85]. The plasma membrane is fluid like with proteins and lipids co-existing within in a heterogeneous distribution. Also, the negative charge on PIP_2_ plays a crucial role in the interaction with membrane bound proteins.

### 4.1. Charge Dependence and Electrostatic Interaction

Over 30 years the electrostatic properties of membranes have been highlighted in the literature [86,87]. Many theoretical models have been proposed based on the smeared charge model of Gouy-Chapman theory, Finite-difference Poisson-Boltzmann (FDPB) method, based on dielectric properties of the solvent [88,89]. Afterwards, it was proposed that flat lipid bilayers can be considered for the electrostatic calculations of the present PIP-based systems when proper choice of orientations are made, concluding that the specific charge of PIP_2_ with respect to the cell membrane is required for lipid signaling events to occur [90]. Effort has been made to understand the atomic level structural of PIP_2_ such as its protonation state and binding to cations, by using hybrid quantum mechanics and molecular mechanics simulation methods which determine the optimal geometry of PIP_2_ [91]. PIP_2_ has high negative charge density obtained by deprotonation of two phosphomonoester groups, which can range from −3e to −5e, depending on pH and the counterions present, which brings the net lipid charge to -3.99 ± 0.10e. The charges on PIP_2_ regulate its interaction with proteins [55].

**Figure 4:**
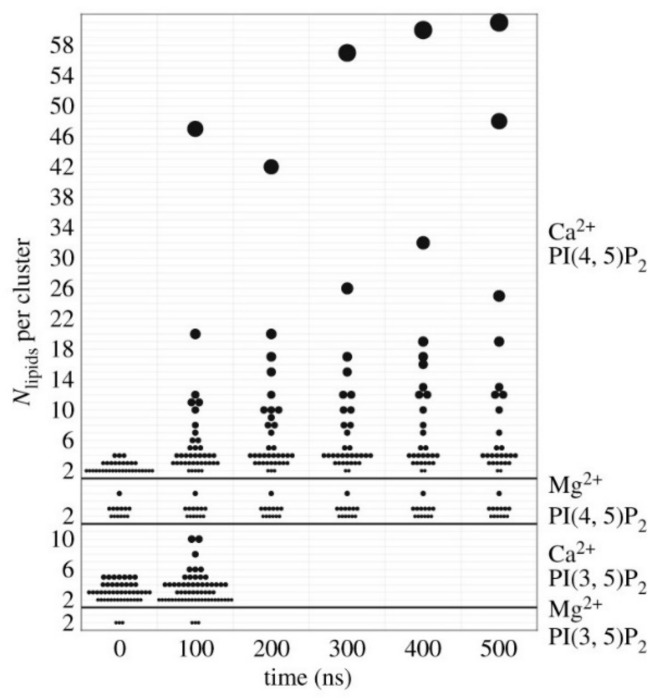
Histograms of cluster of lipids which is also measured on the vertical axis. Only the unique combination of PI(4, 5)P_2_ and Ca^2+^ shows large and growing clusters. The symbol area is proportional to the number of lipids in the cluster (Bradley et al.) [95].

Another important characteristic of, phosphatidylinositol-(4,5)-bisphosphate (PI(4, 5)P_2_) is that the different lateral organizations, such as small clusters or large stable aggregates, which are interconvertible, within the region of the membrane are associated with diverse functionality [92,93]. PI(4, 5)P_2_ turnover at the plasma membrane has been observed by immunofluorescence probes suggesting the evidence of spatially segregated of PI(4, 5)P_2_ pools [93]. Non-homogeneous distribution of PI(4, 5)P_2_ in membranes is due to electrostatic interaction between neighboring lipids [85,94]. Levental et al. showed that of PIP_2_ clustering depends upon the multivalency of the counterion and high charge density of the lipids by using lipid monolayers [93]. Lateral organization on a large range of length scales can be remodeled when Ca^2+^ is introduced to PIP_2_ containing membrane monolayers at different concentrations. This leads to domain formation and reduces phase co-existence surface pressure in of PIP_2_ containing monolayer [93]. The formation of the domains or nano clusters has relevance in cellular function, and is regulated by the Ca^2+^ ions in the absence of proteins [94,95]. Not only Ca^2+^ but other divalent ions such as Mg^2+^ and Zn^2+^ also affect lateral organization of PIP_2_ in the asymmetric membrane at physiological concentration which in turn regulate PIP_2_ protein interaction [94]. Bradley et al. have characterized multivalent lipid cation interaction by the number of lipids bound within a specific distance (called N-bridge), showing the largest cluster formations up to 60 lipids for the combination of PIP_2_ and Ca^2+^ (Figure 4). The formation of clusters is also dependent on physiological trivalent ions such as Fe^3+^ and Al^3+^ [96]. These findings suggest that the electrostatic sequestration and condensation of PIP_2_ by divalent and trivalent ions resulted in increasing the molecular packing and ordering the more disordered phase, which has important biological relevance.

In the lipid bilayer, lateral distribution of PIP_2_ has been affected by both electrostatic interaction and cholesterol dependent phase mixing [50]. The cholesterol enriched region in the membrane forms heterogeneous nanoscale clusters having a size of 10–200 nm, known as lipid rafts which are compartmentalized in the plasma membrane and regulate different cellular functions [84]. Nano clusters of phosphoinositide, localized in the membrane can be visualized by fluorescently labeled pleckstrin homology (PH) domains, which allow PIP_2_ visualization by protein-domain–GFP chimeras in live cells and PLCδ_1_-PH at plasma membrane and OSBP-PH at Golgi membrane; and for PI(3,4)P_2_ visualization with Akt-PH at plasma membrane [36,97].

### 4.2. PIP_2_ Regulation in Membrane Curvature Sensing and Transport

**Figure 5:**
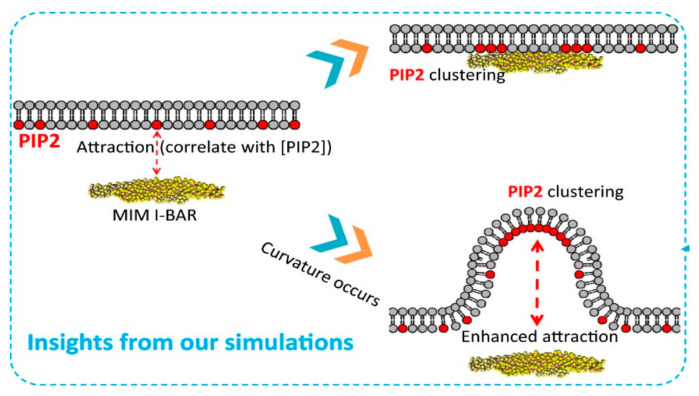
PIP 2 molecules are necessary to recruit MIM I-BAR, which in turn can induce local PIP 2 clustering at its two ends after binding to the membrane (upper panel). Spontaneous bending of lipid membranes can re-distribute PIP 2 molecules to the negatively curved membrane areas (lower panel), which promotes the recruitment of MIM I-BAR and maintaining the curvature [100].

**Figure 6:**
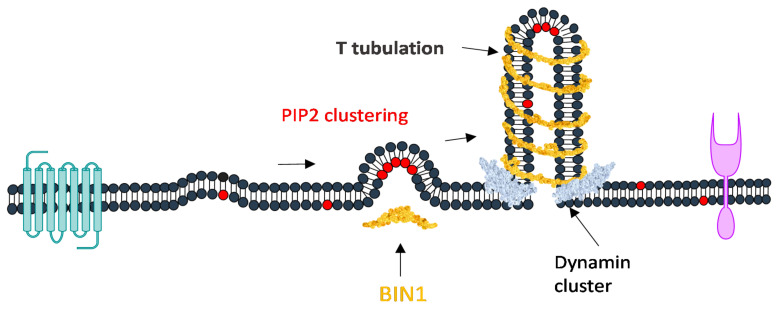
BIN1 mediated membrane tubulation. BAR domain proteins are able to both sense and induce membrane curvature. BIN1 clustering with PIP2 that promote dynamine recruitment and thus forms T- tubule. Binding of dynamin depends upon the amount of PI(4, 5)P_2_ and enhanced by BIN1.

PIP_2_ interacts with many transmembrane proteins such as Bin-Amphiphysin-Rvs (BAR) domain proteins, curvature sensing proteins that are important in regulating membrane shape transitions during endocytosis and membrane trafficking [98,99]. These BAR domain protein interactions with PIP_2_ are charge dependent. By coarse grain modeling Li et al. showed that the electrostatic interaction between PIPs head group which contains large negative charges and many positive charged residues in the BAR is the origin of membrane binding [100]. PIP_2_ binds to both sides of BAR proteins to form membrane protrusion by synergistically binding to actinm [101]. Experimentally and by simulation it has been shown that PIP_2_ has preference in binding to the negatively curved membrane over positively curved membranes (Figure 5) [98]. Thus, membrane curvature can promote the spatial regulation on PIP_2_ binding and local enrichment of lipids. It has been shown in vitro and also in cells that the phosphoinositide binding domain of BIN1 targets the membrane by interacting with PIP_2_.The N-BAR domain of BIN1 clusters with PIP_2_ to promote the recruitment of its downstream partner dynamin and is responsible for membrane tubulation^2^. Amphiphysin1 (BIN1) in PIP_2_-containing membranes induces curvature [102]. Membrane curvature sensing and generation of BIN1 is abrogated in membranes lacking PIP_2_. However, BIN1 alone can initiate membrane tubulation (Figure 6). BIN1 membrane curvature sensing and generation show autoinhibition regulated by downstream ligands and PIP_2_ [102]. A recent study demonstrates that mutation of BIN1 N-BAR impairs membrane T tubulation [103]. This affects the regulation of muscle functioning or nuclear positioning, leading to diseases like centronuclear myopathies [2,104].

PIP_2_ is a major regulator of voltage gated ion channels, in which PIP_2_ binds to the transmembrane domain [20]. Kobayashi et al. showed in skeletal muscle that PIP_2_ is a major activator of Ca^2+^ channels. Depletion of PIP_2_ induces increases in voltage sensitivity and a decrease in voltage amplitude in K^+^ ion channels in *Xenopus* oocytes. PIP_2_ controls both movement and stability of the channels by interacting through linkers [21]. ATP-sensitive K+ channel rundown, the process by which a channel steadily decreases in conductance until the channel inactivates, is induced by Ca^2+^, and this process is shown to be regulated by PIP_2_. KCNQ is another family of channels that absolutely requires PIP_2_. The importance of PIP_2_ in modulating KNCQ channels is well studied in neurons, showing that PIP_2_ hydrolysis increases neuroexcitability and in cardiac arrythmias in patients by showing PIP_2_ dependent channel activation [22,105].

A potentially important event that occurs at the cell surface is the interaction between the lipid bilayer with Ras, a small GTPase and with its effectors. These interactions are shown by molecular dynamics simulation or FRET in live cells. Ras proteins, such as H-Ras, N- Ras, and K-Ras, operate in the inner plasma membrane and are mutated in many cancer types [106]. Recent studies have shown Ras enrichment in nano clusters within phosphatidylserine-rich regions. PIP_2_ binds to Ras G-domain and K-Ras4b HVR, which is one of the isoforms of Ras^3^. Experimental or computational studies showed the tight binding between PI(4, 5)P_2_ and K-Ras4b by measuring rotational dynamics by random amine labeling and by atomic force microscopy. The rotational dynamics of K-Ras are important for signaling in cancer cells [3,107].

## 5. Intracellular Trafficking

PPIs are spatially localized in different compartments in intracellular organelles [7,108]. For example, the Golgi is enriched with PI(3)P or PI(4)P, which are also enriched in early endosomes [109,110]. The Golgi plays a crucial role in membrane trafficking [18,25]. Phosphoinositide 3- kinase converts PI(4, 5)P_2_ to PI(3,4,5)P_3_ which is important for vesicular trafficking [111]. New studies demonstrate that the cell surface membrane is a major site of action for PIP_2_, and the localization of it in different compartments is directly correlated to intracellular trafficking such endocytosis and exocytosis [112]. By specifically interacting with proteins, PIP_2_ controls the formation and spatiotemporal organization of many protein complexes that are involved in intracellular trafficking.

**Figure 7:**
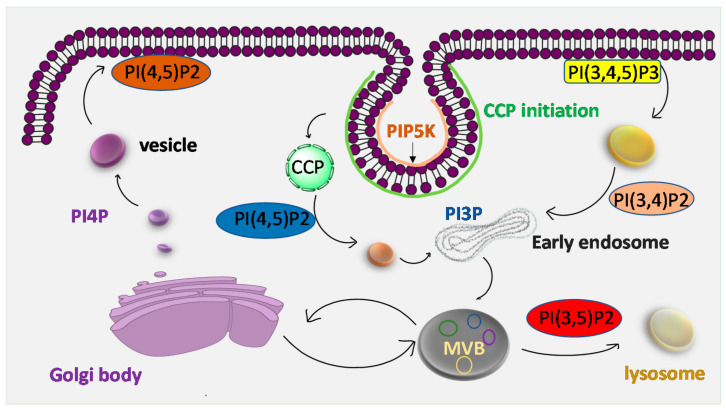
PIP_2_ is involved in intracellular trafficking and vesicular transport. PIP_2_ participates in both clathrin mediated (CCP) and non- clathrin mediated endocytosis. PI(3,5) P_2_ is involved in exocytosis whereas PI(4,5) P_2_ and PI(3,4) P_2_ are involved in endocytic processes.

Clathrin mediated endocytosis, in which cargo is packaged into vesicles with clathrin coating, plays a crucial role in cell signaling, migration and cell-cell interactions. PIP_2_ has been implicated in clathrin-mediated endocytosis [113]. However, clathrin does not directly bind to the membrane or cargos but to adaptor proteins such as adaptor protein 2 (AP2) or accessory protein AP180 and espin [114]. A recent study reported that during clathrin coated pit (CCP) assembly initiation AP2 is recruited to the plasma membrane and colocalizes with the nucleation complex which binds to both cargo and PIP_2_, when stained with anti- PIP_2_ antibody^111^. The formation of PI(3,4)P_2_ by class II PI3-kinase C2α (PI(3)K C2α) spatiotemporally controls clathrin-mediated endocytosis. The depletion of PI(3,4)P_2_ hinders the maturation of CCPs before fission. PIP5K is associated with the initiation of CCPs but its activity is not found to mature them. Another study shows that PIP_2_ is an established regulator of endocytosis. Endosomal PI(4,5)P_2_ is required for the sorting of active epidermal growth factor receptor (EGFR) towards multivesicular bodies (MVB) and further termination of the signal (Figure 7). Sun et al. showed that type I gamma phosphatidylinositol phosphate kinase (PIPKIγ) is an enzyme that synthesizes PIP_2_ by phosphorylation of PtdIns4P and regulates EGFR sorting from endosomes to lysosomes [115]. This was done by performing flow cytometry analysis and quantification of internalized Alexa Fluor 488-labelled EGF in control and PIPIγi5- knockdown cells [111,115]. PIPIγi5 interacts with sorting nexin 5 (ANX5) which is the effector of PIP_2_ in the early endosome, but cells lacking SNX5 still localize PIPIγi5 to endosomes [116]. SNX5 has been reported to inhibit EGFR degradation when overexpressed. However, knockdown of SNX5 does not affect EGFR trafficking to early endosomes, but blocks trafficking to the late endosome/lysosome [115,117]. On the other hand, EGFR regulates Ras activity, which is implicated in PIP3 and MAP kinase pathways [118].

Another crucial role of PIP_2_ is in bidirectional homeoprotein trafficking. Homeoproteins are a class of transcription factor that predominantly resides in the nucleus. Chick engrailed 2 (EN2) is a homeoprotein that shuttles between the nucleus and cytosol. In the cytosol, EN2 associates with those membrane fractions enriched in cholesterol and glycosphingolipids. EN2 directly binds to PIP_2_. Dephosphorylation of PIP_2_ reduces EN2 secretion. Moreover, PIP_2_ is involved in EN2 internalization [119].

Phosphoinositides are interconvertible and the balance of production and usage is tightly controlled in a specific organelle. Contrary to the plasma membrane, the Golgi membrane has less PIP_2_ and high abundance of PI4P and PI4K enzymes [1,120,121,122]. There is a possibility that the plasma membrane PI4P pool is due to vesicular trafficking of PI4P from Golgi membrane. The recent discovery of lipid binding domains enables the lifetime monitoring of lipid synthesis by fusing with green fluorescent protein GFP. GFP-tagged PKB/Akt PH and GFP-PH (PLCδ) are possible markers for live monitoring PIP_3_ and PIP_2_. Utilizing recent advancements, a study demonstrated PIP_2_ level decreases in the plasma membrane when stimulated by angiotensin II (AngII) by showing the change in PLCδ_1_PH-GFP expression level in HEK-293 cells and an increase of GFP in cytosol [123,124]. Recovery experiment shows that Golgi PI4P eliminated cells recover slowly compare to control. These studies confirm that although PI4P takes part in the maintenance of the PIP_2_ level pool at plasma membrane, it is not requisite for the process [109].

Phosphatidylinositol 3,5-bisphosphate PI(3,5)P_2_ is synthesized from PI3P by FYVE-domain-containing PI kinase (PIKfyve) in mammalian cells [125]. FYVE domain appears to target the enzyme to PI3P -rich membranes [126]. However, a similar process occurs in yeast called *Saccharomyces cerevisiae*, and the PI(3,5)P_2_ synthesis is found to be processed by Fab1p. Since Fab1 is not responsible for the full synthesis, additional unknown effector proteins are expected to be involved. PI(3,5)P_2_ is involved in vacuole to lysosome membrane trafficking and packaging of proteins in multivesicular bodies (MVBs) [1,127].

## 6. PIP_2_ in Signaling and Diseases

Accumulating evidence suggests that PIP_2_ dysregulation contributes to cancer including melanoma, breast cancer, leukemia, prostate cancer (Table 1) [128,129]. Literature suggests that PIP_2_ is implicated in many pathways and binds to signaling proteins such as lamellipodin/RAPH1, tandem PH domain-containing proteins TPP1 and TAPP2 and PIP3 binding proteins including protein kinase Akt/PKB [59,130]. The PIP3-Akt signaling pathway is implicated in many diseases [130,131].

**Figure 8:**
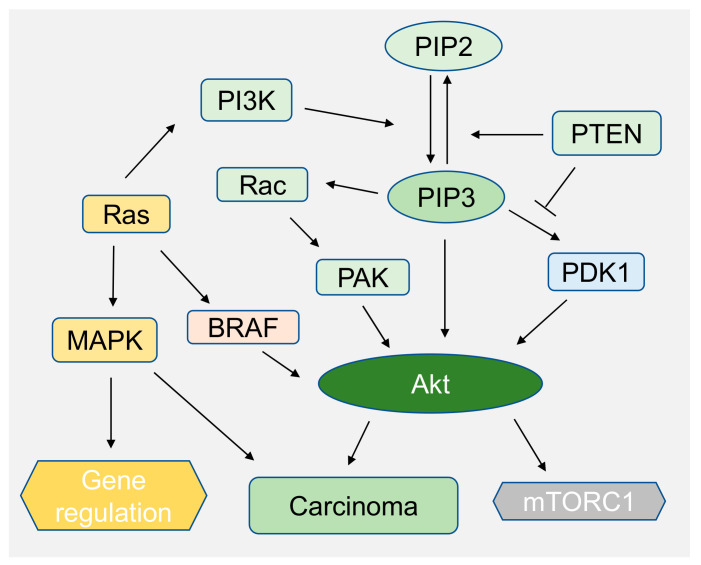
PIP_3_ and MAP kinase pathways synergistically and independently regulates melanoma cancer or any other carcinoma. Ras regulates both PI3 and Akt kinase pathways. In addition, Ras independently regulates BRAF which is also implicated in Akt3 activity depicted in diagram.

Phosphoinositides play a major role in intracellular signaling pathways which are implicated in carcinogenesis such as hepatocellular carcinoma (HCC) or melanoma [33,128,139,140]. Thus, many signaling pathways are targeted for therapies including phosphoinositide 3-kinase (PI3K)/Akt, mitogen-activated protein kinase (MAPK) pathways [141]. Numerous proteins are regulated downstream of these pathways. Generally, pathways are activated by the alteration of the cell’s microenvironment or genetic alteration [33]. Our recent work shows that, when Huh7 cells, a hepatocellular carcinoma cell line, adhere to soft hyaluronic acid (HA) gels, they show similar behavior as cells adhered on stiff polyacrylamide gels by regulating phosphoinositide signaling. The result is confirmed by pAkt expression level by immunoblotting and by quantifying the total amount of PIP_3_ on HA and Poly acrylic acid (PAA) substrates by using mCherry Grp1, a fluorescent protein that specifically binds to PIP_3_ [33,142]. PIP_3_ which increases Akt activity via PDK1, is activated by Ras (Figure 8). PDK1 activates Akt by phosphorylation of threonine site. The overexpression of PAK, which is one of the downstream effectors of PIP3, is correlated with many cancer types such as ovarian cancer [15,106]. PI3K catalyzes PIP_2_ into PIP_3_ [143] (Figure 8). One of the major downstream effectors of Akt is mTORc1 which is deregulated in many cancers when phosphatase and tensin homolog deleted on chromosome 10 (PTEN) gene dephosphorylates PIP_3_ to PIP_2_ (Figure 8) [15].

The PIP3-Akt pathway also synergistically activates MAPK signaling pathway in melanoma cancer development [118]. The MAPK pathway is one of the most investigated signaling pathways in melanoma cancer [143]. Thus, a series of inhibitors for these pathways are targeted for the therapeutics of melanoma. It has been observed that PI3K activity is increased in melanoma due to loss of PTEN (Figure 8) or increased levels of Akt3 activity, and that plays a crucial role in early melanoma development. A recent study has shown that Akt3 phosphorylates B-Raf (Figure 8) which is often mutated in melanoma cancer [118]. Lipid binding domains such as PH domains of Akt/PKB, are important in signaling, which depends upon PIP synthesis. Akt/PKB binds to PI(3,4)P_2_ and PIP_3_ to regulate cell survival and growth, which is independent of PI(4,5)P_2_. In cases of melanoma, 50% of patients progress to metastatic stress due to upregulation of protein tyrosine phosphate (PTP) promoting cell migration and invasiveness [139]. PTPs bear phosphatase activity toward lipidic substrates, including phosphoinositides. PRL-3 is one of the dual specificity phosphatases which is associated with intracellular membranes and cellular migration [129]. PRL-3 dephosphorylates PI(4.5)P_2_ and thus alters the phosphoinositide level in cells [144]. PRLs are overexpressed in many cancer types and have become the target of many cancer therapies including melanoma [129,139,145].

ORCL (oculo-cerebro-renal syndrome of Lowe) enzymatic activity is found in many compartments in cells especially concentrated at the Golgi network. The mutation of ORCL causes oculo-cerebro-renal syndrome of Lowe which is an X-linked condition [133]. Lowe syndrome leads to many diseases including renal Fancomi syndrome, glaucoma, cataracts, blindness, mental retardation (Table 1). ORCL is a key component of endocytic trafficking which is involved in clathrin coated pits and other binding motifs, such as AP2, APPL1, and Rab GTPase, including Rab5, Rab6, and Rab14. Therefore, inactivation of ORCL leading to deregulation of PI(4,5)P_2_ level influences trans- Golgi network and endosomal activity. Imbalance of PIP_2_ levels further affects actin dynamics and actin binding proteins. Moreover, ORCL controls reabsorption of proteins via PI(4,5)—phosphatase in renal proximal tubule cells (PTCs) [134]. Another regulator for Down’s syndrome is synaptojanin1 which acts on both PI(4,5)P_2_ and PI(3,5)P_2_, found in endocytic intermediate nerve terminals. Synaptojanin regulates the actin pool, as well as the de-coating of cathrin mediated endocytic vesicles and synaptic vesicles. In synaptojanin deficient mice, the PI(4,5)P_2_ level increases whereas a decreased level of cytosolic inositol 5 phosphatases in neurons is observed. Also, an increase in clathrin coated vesicles in nerve terminals is observed [135]. Inositol polyphosphate-4-phosphatase (INPP_4_) which binds to PI(4,5)P_2_, shows a reduced level in an asthma mouse model due to restrictive stress [130]. Oxidative stress which is generated by reactive oxygen species (ROS) stimulates the accumulation of PI(4,5)P_2_. ROS has been implicated in airway inflammation. INPP_4_ deficiency also leads to cancer, including breast cancer and neurodegeneration [131].

The mutation of myotubularin (MTM) causes several disorders such as failure of skeletal muscle development. MTM related proteins, MTMR1-13 which is an inactive partner of MTMR2 causes the same mutation as active member. Each MTM protein regulates a specific pool of PI(3)P and PI(3,5)P_2_. Another disease where PI(4,5)P_2_ regulation is important involves the human immunodeficiency virus-1 (HIV-1). Viral entry into the host cell requires actin cytoskeletal reorganization. Viral receptor clustering is regulated by actin adaptor proteins, such as moesin, filamin A, gelsolin, tailn, vinculin, profilin, WASP, and Arp2/3, that are controlled by PI(4,5)P_2_. PI(4,5)P_2_ production is regulated by HIV-1 attachment and promotes viral infection. Hence, the virus controls actin dynamics during cycle, by facilitating actin polymerization and depolymerization [137]. In HIV-1 infection, CD4 and coreceptors clustering at the cell surface is induced by glycoprotein g120, which facilitates virus envelope and cell membrane fusion. PI(4,5)P_2_ is required to recruit the gag protein at the cell membrane to facilitate invasion. A high density of PI(4,5)P_2_ is not only required for HIV-1 recruitment but also to maintain glycoprotein at the membrane [136]. PIP_2_ plays a central role in many neuronal and synaptic functions by regulating endocytosis, exocytosis, cytoskeletal reorganization, and ion channels. In Alzheimer’s disease, Amyloid- β peptide(Aβ) oligomers cause impairment of synaptic function. Elevation of Aβ in the Alzheimer’s diseased brain results in decreased levels of PI(4,5)P_2_ [138].

## 7. Conclusions

This review summarizes the role of PIP_2_ and other PPIs in cell membrane dynamics, focal adhesion, actin organization, intracellular signaling and disease. PIP_2_ regulates actin binding protein activity which either promotes polymerization and depolymerization of actin filament. Past evidence suggests that actin is connected to the membrane via actin binding proteins such as α-actinin or filamin which are regulated by phosphoinositides. These interactions also affect the binding of actin filaments with focal adhesion proteins such as paxillin, talin, FAK, or vinculin. The distribution of PIP_2_ in the membrane regulates cell signaling. PIP_2_ activity depends upon the concentration of cholesterol and divalent ions such as Ca^2+^, Mg^2+^, or Zn^2+^. In addition, PIP_2_ plays a crucial role in modulating many signaling pathways such as PIP3/Akt, mTORc1, or Rho dependent pathways that have implications in many diseases including cancer, neurodegenerative disease, or down syndrome.

Although PPIs are essential for many cellular functions, there are disparities in many processes which need further studies. PIP_2_ plays an important role in actin reorganization and filament dynamics. However, the role of PIP_2_ in any other cytoskeletal component has not yet been well studied. Among PIP_2_ binding actin proteins, LIMK1 and LIMK2 play an overlapping role in actin reorganization in the Rho-ROCK pathway. Further studies are required to differentiate the functional role of LIMK1 and LIMK2. Moreover, it is unclear if members of ROCK and PAK family proteins function as LIMK- activating kinases. Cortactin shows dependencies on PIP_2_ and Rac in dissociating from actin–myosin complex, although the direct implication of PIP_2_ in regulating cortactin still remains controversial [146], and other activators such as the endocytic protein Abp1p remain unclear. It has been shown that a synthetic peptide of α-actinin inhibits PLC-γ1 and PLC-δ1. It is ambiguous whether PIP_2_ bound to α-actinin is hydrolyzed by activated PLC-γ1 or not. The interaction of vinculin and membrane is based upon either full length or tail domain of vinculin in lipid bilayers or in cells. However, a specific lipid binding site has yet to be discovered.

## Figures and Tables

**Table 1:** PIP_2_ and enzymatic activity in different pathways in disease [122].

Phospho-Inositides	Pathways/Functions	Enzymatic Activity	Disease Implication	References
PIP3, PI(4,5) P_2_, PI(3,5) P_2_	PI3K-Akt	PI3K, PTEN 1,2	Melanoma cancer, Cowden disease, pancreatic cancer, ovarian cancer.	[132]
PI(4,5) P_2_	Endocytic trafficking pathways	OCRL, 5 phosphatases	Oculo-cerebro-renal syndrome of Lowe: renal Fancomi syndrome, glaucoma, cataracts, blindness, mental retardation.	[133,134]
PI(3,5) P_2_		MTM1, PI4P,	Myopathy.	[2,121]
PI(3,5) P_2_		Fab1/PIKfyve kinase	Neuropathologies, Charcot-Marie tooth disease.	
PI(4,5) P_2_, PI(3,5) P_2_	Endocytic pathways	Synaptojanin1,2	Bipolar disorder, Down syndrome, neuronal disorder.	[29,85,135]
PI(4,5) P_2_		INPP_4_	Asthma, nondegeneracy.	[23,130]
PI(4,5) P_2_	Actin reorganization		Human immunodeficiency virus-1 (HIV-1).	[136,137]
PI(4,5) P_2_	impairment of synaptic function	Amyloid-β peptide oligomers	Alzheimer’s disease	[138]
PI(3,4) P_2_. PIP3	Akt/PKB		Cell survival and growth, cancer.	[16,130,131]
PI(4,5) P_2_		PRL-3	Melanoma, colon cancer.	[131,136,137]
